# Supplementary material for: Thermal stability and coalescence dynamics of exsolved metal nanoparticles at charged perovskite surfaces
Source: Nat Commun. 2024 Nov 9;15:9724. doi: 10.1038/s41467-024-54008-4 (PMC11550403; doi:10.1038/s41467-024-54008-4)
Supplement: Supplementary file 1 — Supplementary Information [file 41467_2024_54008_MOESM1_ESM.pdf]

Supplementary Information

Thermal stability and coalescence dynamics of exsolved metal nanoparticles at charged perovskite surfaces

Moritz L. Weber[*,1,2,3,4], Dylan Jennings[4,5], Sarah Fearn[2], Andrea Cavallaro[2,6], Michal Prochazka[1,7], Alexander Gutsche[3], Lisa Heymann[3], Jia Guo[2], Liam Yasin[8], Samuel J. Cooper[9], Joachim Mayer[5,10], Wolfgang Rheinheimer[4,11], Regina Dittmann[3], Rainer Waser[3,12], Olivier Guillon[4,13,14], Christian Lenser[4], Stephen J. Skinner[2], Ainara Aguadero[2,15], Slavomir Nemšák[*,1,16] and Felix Gunkel[*,3]

[1]*Advanced Light Source, Lawrence Berkeley National Laboratory, Berkeley, California 94720, United States*

[2]*Department of Materials, Imperial College London, Exhibition Road, London SW7 2AZ, United Kingdom*

[3]*Peter Gruenberg Institute for Electronic Materials (PGI-7) and Juelich-Aachen Research Alliance (JARA-FIT), Forschungszentrum Juelich GmbH, 52425 Juelich, Germany*

[4]*Institute of Energy and Climate Research for Materials Synthesis and Processing (IEK-1), Forschungszentrum Juelich GmbH, 52425 Juelich, Germany*

[5]*Ernst Ruska-Centre for Microscopy and Spectroscopy with Electrons, Materials Science and Technology (ER-C 2), Forschungszentrum Juelich GmbH, 52425 Juelich, Germany*

[6]*Department of Engineering and Applied Sciences, University of Bergamo, 24044 Dalmine, Italy*

[7]*University of West Bohemia, New Technologies Research Centre, 301 00 Pilsen, Czech Republic*

[8]*Faculty of Engineering, Department of Materials, Imperial College London, Exhibition Road, London SW7 2AZ, United Kingdom*

[9]*Faculty of Engineering, Dyson School of Design Engineering, Imperial College London, Exhibition Road, London SW7 2AZ, United Kingdom*

[10]*Central Facility for Electron Microscopy (GFE), RWTH Aachen University, 52064 Aachen, Germany*

[11]*Universität Stuttgart, Institute for Manufacturing Technologies of Ceramic Components and Composites (IMTCCC), Stuttgart 70569, Germany*

[12]*Institute for Electronic Materials (IWE 2), RWTH Aachen University, 52074 Aachen, Germany*

[13]*Institute of Mineral Engineering (GHI), RWTH Aachen University, 52062 Aachen, Germany*

[14]*Juelich-Aachen Research Alliance (JARA-Energy), 52425 Juelich, Germany*

[15]*Instituto de Ciencia de Materiales de Madrid (ICMM-CSIC), Sor Juana Ines de la Cruz 3, 28049, Madrid, Spain*

[16]*Department of Physics and Astronomy, University of California, Davis, California 95616, United States*

E-mail: mo.weber@fz-juelich.de; SNemsak@lbl.gov; f.gunkel@fz-juelich.de

Supplementary Note 1: Thin film synthesis and characterization

Epitaxial STNNi (Fig. S1) and STNi (Fig. S2) thin films can be synthesized with high quality. RHEED monitoring reveals a layer-by-layer growth mode for the two materials (Fig. S1a and (Fig. S2a). All films with thicknesses up to 100 nm exhibit a stepped surface morphology with sub-nanometre roughness (Fig. S1b and (Fig. S2b), while the surface roughness of STNNi and STNi thin films slightly increases with increasing thin film thickness. STNi thin films (RMS 258±89 pm - 798±52 pm) typically exhibit a slightly increased surface roughness relative to STNNi thin films (RMS 151±15 pm - 282±21 pm). Both STNNi and STNi have a slightly larger lattice parameter compared to undoped STO substrate, as visible from the thin film diffraction peaks at lower diffraction angles as compared to the substrate (Fig. S1c and (Fig. S2c). Hence epitaxial deposition results in a minor compressive strain for both materials, which is slightly increased for STNNi due to co-doping with Nb of larger ionic radius compared to the Ti host cations[1]. Reciprocal space mapping reveals in-plane *a*-lattice parameters equal to the STO substrate confirming that the epitaxial thin films grow fully strained (Fig. S1d and (Fig. S2d). High-resolution transmission electron microscopy reveals a well-ordered and coherent perovskite lattice, while a faint contrast becomes visible in the *Z*-contrast imaging. Here, coupled energy dispersive X-ray spectroscopy mapping of the elemental distribution shows small inhomogeneities in the Ni dopant distribution, where the accumulation of dopants becomes apparent in designated areas of the perovskite host lattice (Fig. S1e) and (Fig. S2e). For more details on dopant inhomogeneities in STNNi, we refer to reference[2]. Our present analysis shows that similar inhomogeneities are also present in STNi.

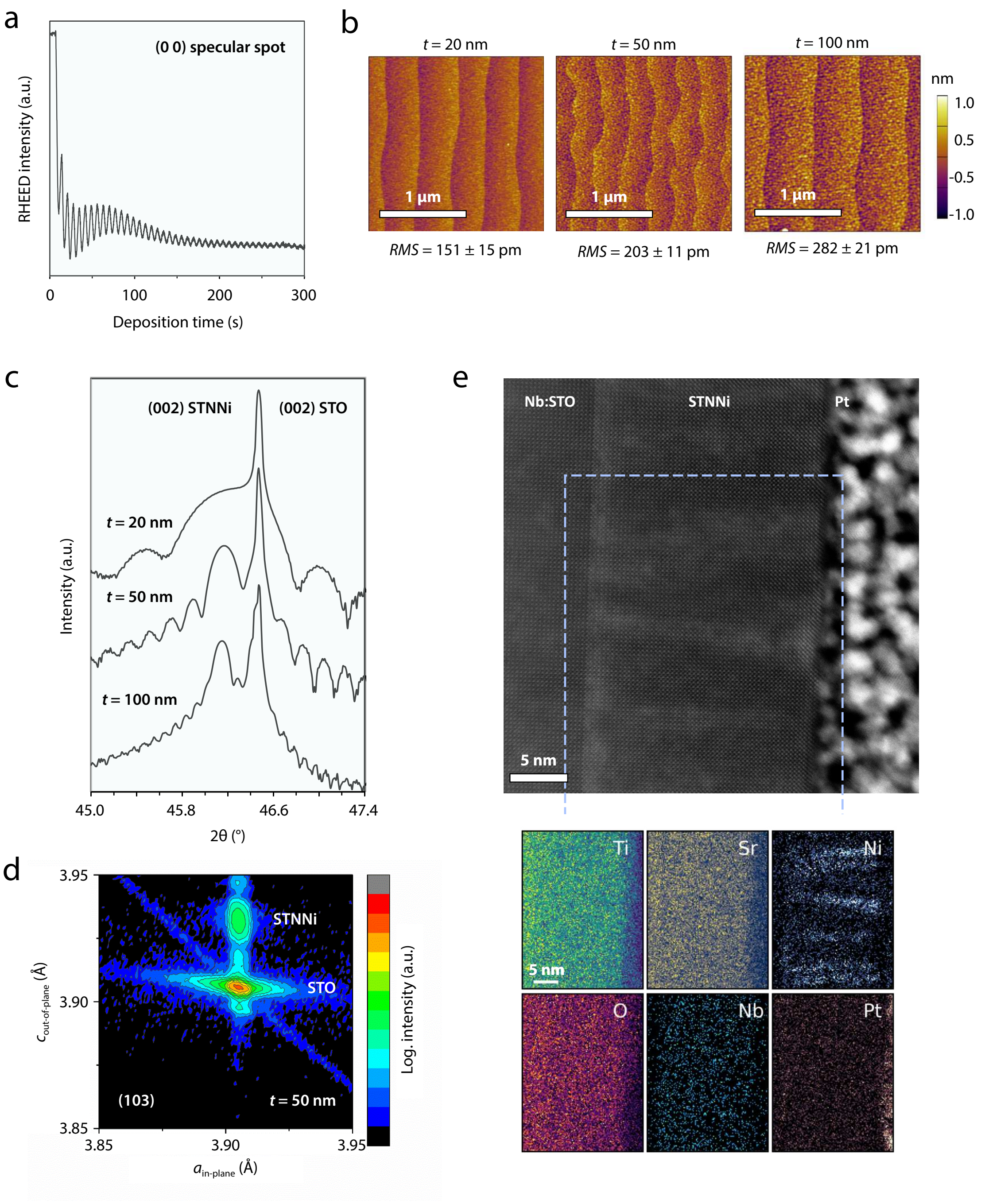

Figure S1: Characterization of the growth mode, surface morphology and crystallographic properties of epitaxial STNNi thin films deposited on TiO_2-terminated (001) $SrTiO_3$ single crystal substrates. (a) In-situ RHEED intensity evolution for the initial growth of STNNi. (b) Thickness-dependent surface morphology of STNNi thin films. The root mean square (RMS) roughness is given below, where the error is the standard deviation of measurements obtained from two thin films that were deposited under equal conditions. (c) Thickness-dependent x-ray diffractograms obtained from STNNi thin films in $2\Theta - \omega$ geometry. (d) Reciprocal space mapping around the (103) diffraction peak. (e) High-resolution transmission electron microscopy and EDXS analysis of a FIB lamella prepared from a representative 20 nm thick as-prepared STNNi sample in cross-section geometry. Bright features in the *Z*-contrast image are correlated to Ni-rich nanostructures indicating an inhomogeneous distribution of reducible Ni dopants.

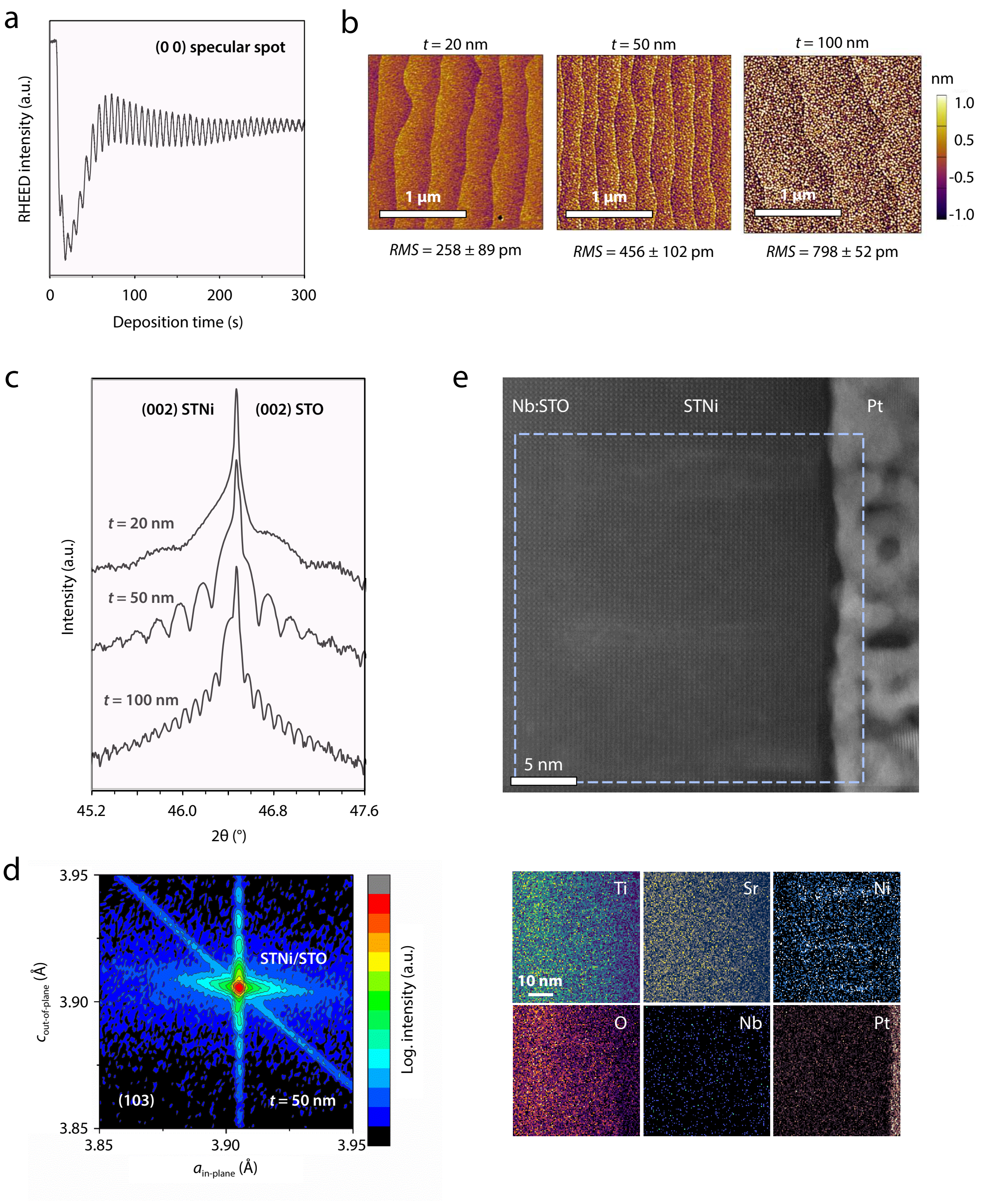

Figure S2: Characterization of the growth mode, surface morphology and crystallographic properties of epitaxial STNi thin films deposited on TiO_2-terminated (001) $SrTiO_3$ single crystal substrates. (a) In-situ RHEED intensity evolution for the initial growth of STNi. (b) Thickness-dependent surface morphology of STNi thin films. The root mean square (RMS) roughness is given below, where the error is the standard deviation of measurements obtained from two thin films that were deposited under equal conditions. (c) Thickness-dependent x-ray diffractograms obtained from STNi thin films in $2\Theta - \omega$ geometry. (d) Reciprocal space mapping around the (103) diffraction peak. (e) High-resolution transmission electron microscopy and EDXS analysis of a FIB lamella prepared from a representative 20 nm thick as-prepared STNi sample in cross-section geometry. Bright features in the Z-contrast image are correlated to Ni-rich nanostructures indicating an inhomogeneous distribution of reducible Ni dopants.

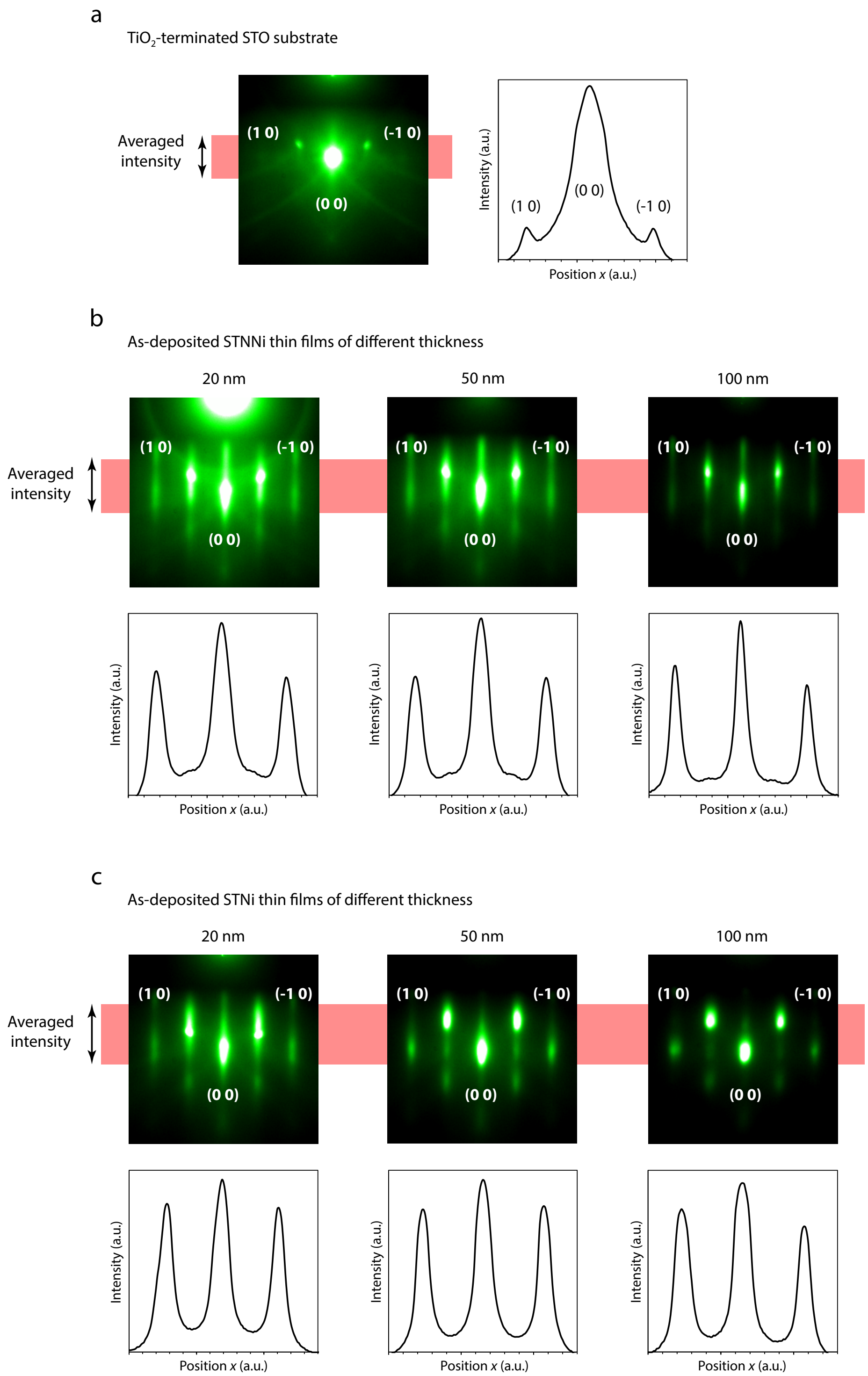

Figure S3: RHEED surface diffraction patterns and line profiles extracted from the the respective diffraction patterns across the (0 0) specular spot and the (1 0), (-1 0) diffraction spots. Representative RHEED diffraction patterns are shown for (a) a TiO_2-terminated STO substrate, (b) for as-deposited STNNi with thicknesses between 20 - 100 nm (c) and for as-deposited STNi thin films with thicknesses between 20 - 100 nm. The horizontal red bars indicate the approximate width of averaged intensity that was applied to extract the line profiles. Note that the absolute intensity of the RHEED patterns may vary due to differences in the primary electron beam alignment.

Fig. S3 shows representative RHEED surface diffraction patterns recorded for as-deposited STNNi and STNi thin films. In addition, the corresponding line profiles that were extracted across the (0 0) specular spot and the (1 0), (-1 0) diffraction signals are given for each diffraction pattern. A representative RHEED surface diffraction pattern obtained from a TiO_2-terminated STO substrate is shown for comparison. All surface diffraction patterns exhibit a predominant 2D-character. For STNi thin films a minor 3D-like pattern becomes apparent for larger thin film thicknesses, which is consistent with a slightly increased surface roughness in the upper sub-nanometre range (cf. Fig, S2).

The intensity of the (0 0) specular spot relative to the (1 0), (-1 0) diffraction spots can provide information about the predominant molecular surface termination[3–6]. For STO a high intensity of the (0 0) specular spot relative to low-intensity (1 0), (-1 0) diffraction signals indicates predominant TiO_2 surface termination as can be seen for the TiO_2-terminated STO substrate that was obtained by chemical etching steps and subsequent thermal annealing (cf. Fig. S3a). After pulsed laser deposition of STNNi and STNi an increased intensity of the (1 0), (-1 0) diffraction signals is detected for both materials. However, the (0 0) specular spot remains the signal of highest intensity, indicating a mixed SrO / TiO_2 surface termination[3,4].

Supplementary Note 2: Surface morphological evolution

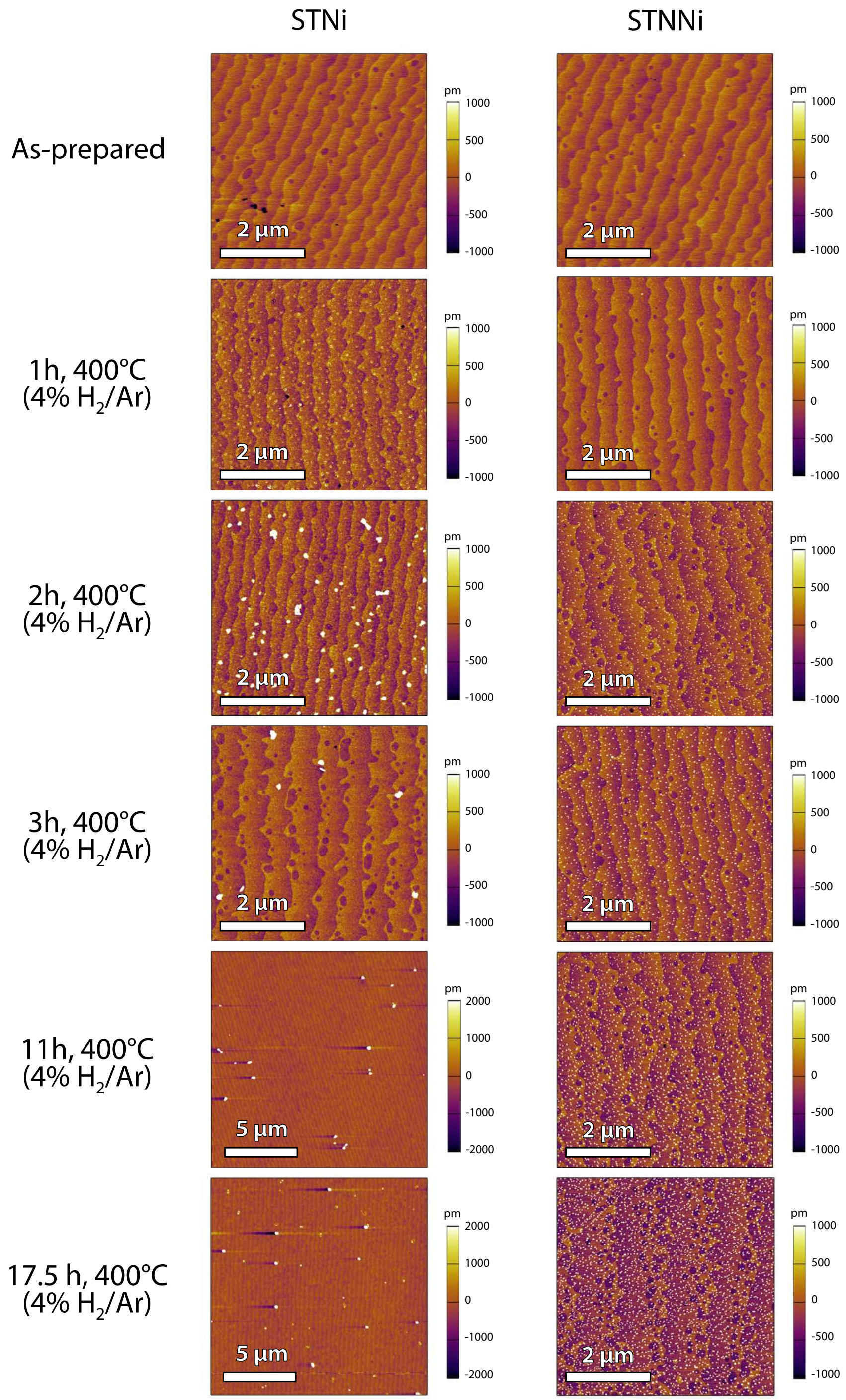

Figure S4: Surface morphology of 20 nm thick STNi and STNNi thin films after joint reduction ($T = 400°C$, 4% H_2/Ar) for different annealing times. Note that images of different scan sizes are shown to take pronounced nanoparticle coarsening at the STNi surface into account.

Fig. S4 shows representative images of the STNNi and STNi surface morphology obtained in the as-prepared state of the thin films and for thin films after thermal reduction with different reduction times ($T = 400°C$, 4% H_2/Ar). As can be seen, STNi exhibits a faster exsolution reaction compared to STNNi, where the formation of a nanostructured surface is apparent already after $t = 1$ h of thermal reduction. At the same time, the exsolved nanoparticles exhibit a fast coalescence behaviour at the STNi surface while remaining finely dispersed at the STNNi surface. Note that the lateral scale and the height scale may vary to account for the different particle distribution and density (larger scan size for STNi after $t = 11$ h and $t = 17.5$ h reduction).

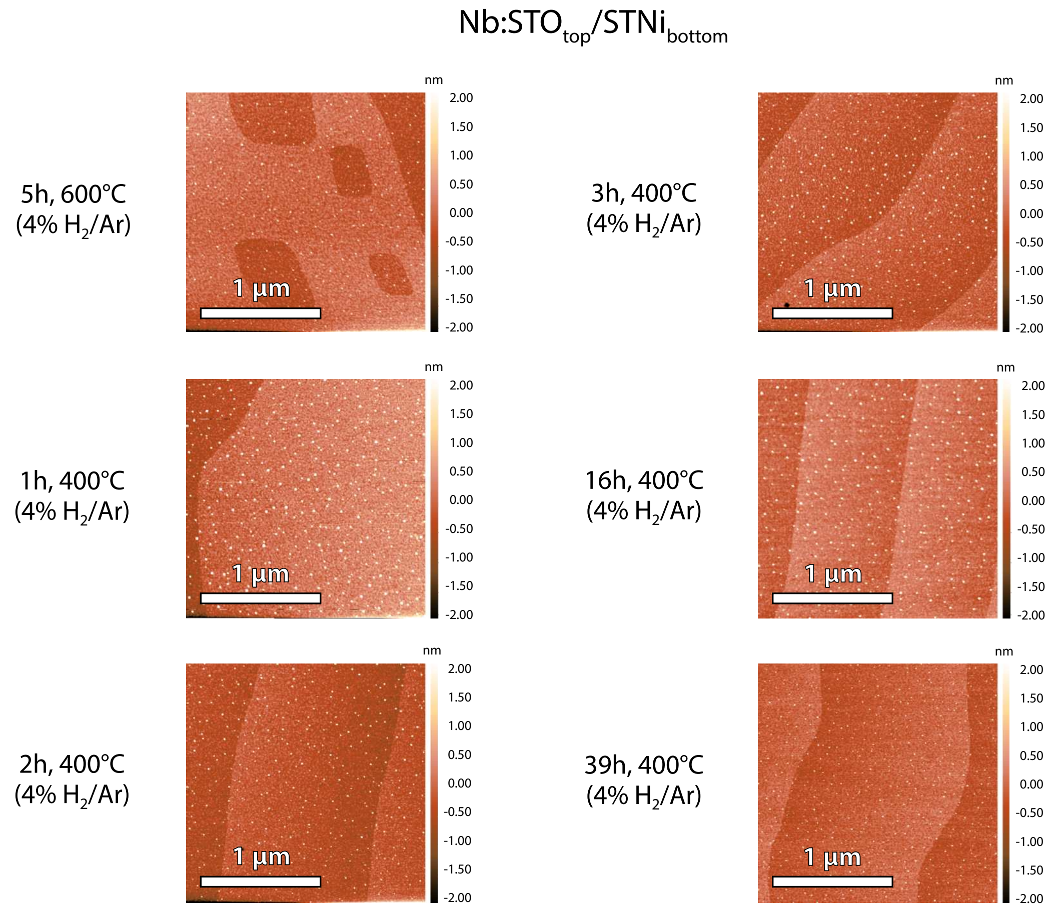

Figure S5: Surface morphology of Nb:STO (4 monolayers) / STNi (20 nm) sample deposited in stack geometry after consecutive thermal reduction steps. The initial nanoparticle population at the surface is fabricated by a reduction step at $T = 600°C$ while the coarsening behaviour is investigated during thermal reduction at $T = 400°$ with different annealing times (4% H_2/Ar).

Fig. S5 shows the surface morphology of a Nb:STO (4 monolayers) / STNi (20 nm) sample deposited in stack geometry after thermal reduction at different conditions. The initial nanoparticle population at the surface is exsolved upon thermal reduction at $T = 600°C$ for $t = 5$ h (4% H_2/Ar). Notably, an initial reduction step at lower temperatures ($T = 400°C$, $t = 50$ h, 4% H_2/Ar) did not result in significant exsolution of metal nanoparticles. Subsequently, several consecutive reduction steps are conducted at $T = 400°C$ (4% H_2/Ar) to investigate the thermal stability of the nanoparticles. The exsolved nanoparticles appear to be robust against coalescence, while high surface mobility of the nanoparticles similar to STNi appears to be suppressed. We presume that fabricating an donor-type surface defect chemistry by epitaxial surface engineering of the exsolution-active acceptor-type STNi thin film results in a stabilization of the exsolved nanoparticles by considerably decreasing the concentration of oxygen vacancies in the surface region of the sample.

Supplementary Note 3: STEM / EDXS analysis of STNNi

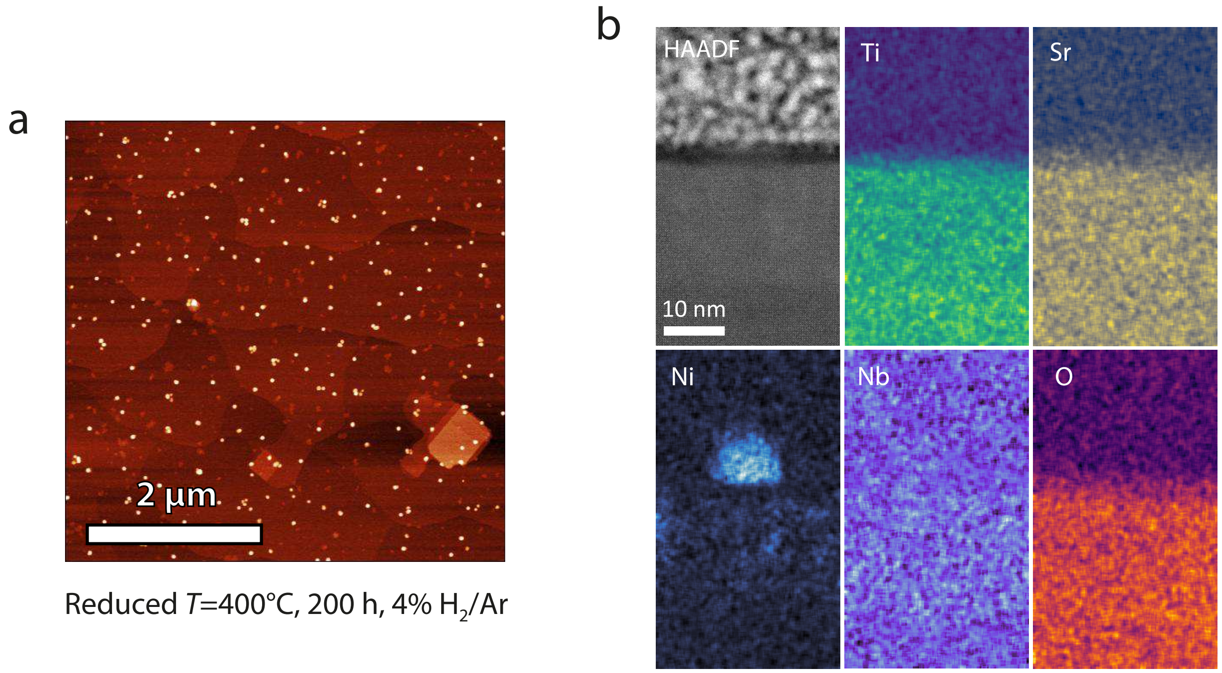

Reduced T=400°C, 200 h, 4% H_2/Ar

Reduced T=900°C, 5 h, 4% H_2/Ar

Figure S6: (a) Atomic force microscopy of the surface morphology of an STNNi thin film after long-term reduction at $T = 400°C$ ($t = 200$ h, $p(4\%\ H_2/Ar) = 1$ bar). The sample shown is equal to the sample investigated by STEM / EDXS as discussed in Fig. 2e of the main manuscript. (b) STEM / EDXS analysis of the nanoparticle-support interface of exsolved nanoparticles at the STNNi surface after thermal reduction at $T = 900°C$ for $t = 5$ h in continuous flow of a $p(4\%\ H_2/Ar) = 1$ bar gas mixture. Independent from the reduction temperature, no indications for nanoparticle socketing were detected.

The surface morphology of STNNi after long-term reduction is shown in Fig. S6a. As can be seen, the size of the exsolved nanoparticles remains relatively small and dispersed across the thin film surface even after 200 hours of thermal reduction. Occasionally, the formation of small nanoparticle clusters can be observed.

Fig. S6b shows scanning transmission electron microscopy (STEM) imaging and X-ray energy dispersive spectroscopy (EDXS) STNNi in cross-section geometry after thermal reduction at $T = 900°C$ for $t = 5$ h ($p(4\%\ H_2/Ar) = 1$ bar). Similar to the sample that was thermally reduced for 200 h as shown in Fig. 2e of the main manuscript, no indications for nanoparticle socketing are detected.

Supplementary Note 4: Ambient-pressure XPS analysis

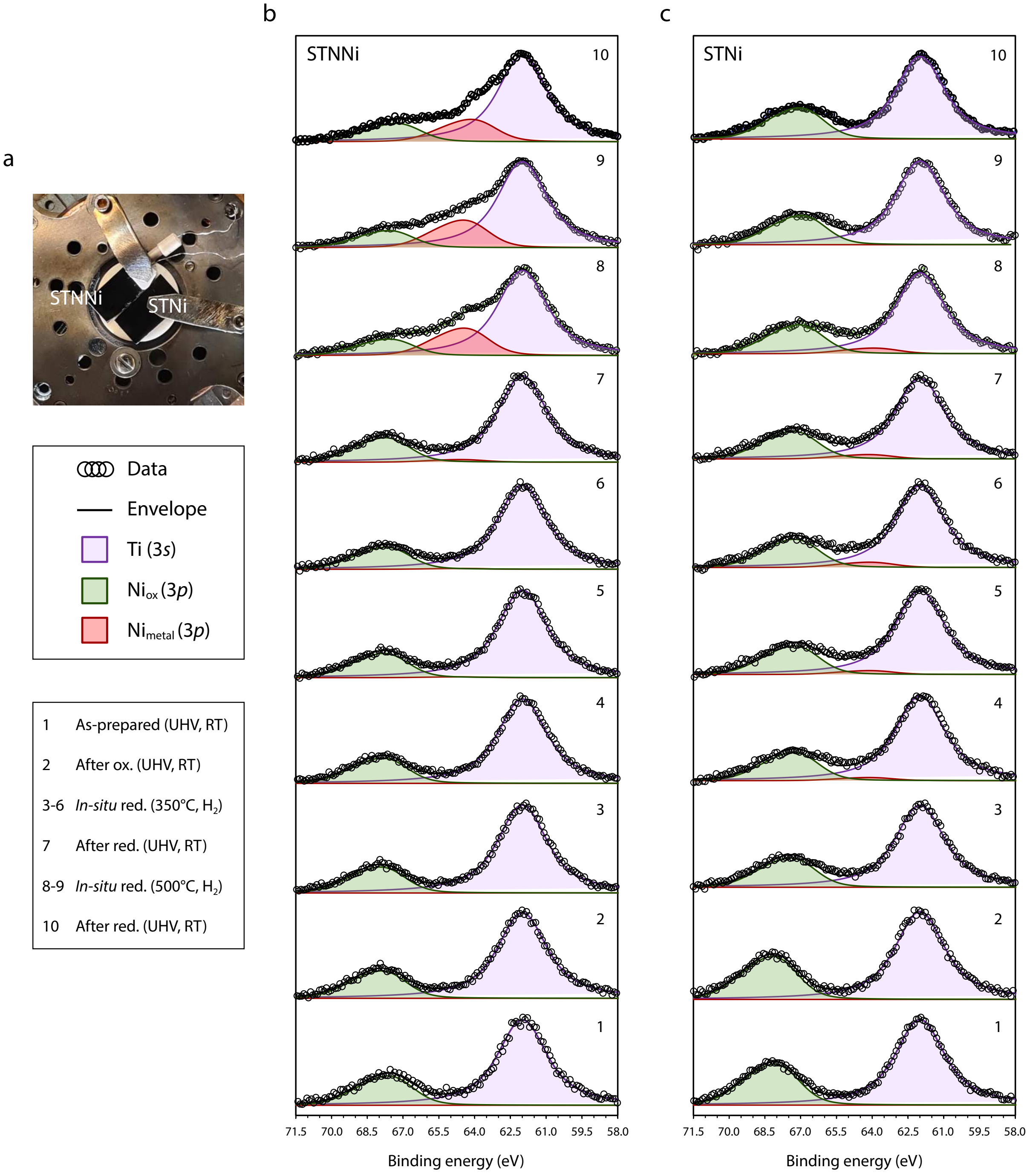

Figure S7: Ambient-pressure X-ray photoelectron spectroscopy. The probing geometry is shown in (a). (b, c) shows the deconvolution of the XPS Ni 3p-Ti 3s core-level spectra by peak fitting. The spectra were obtained from representative STNNi and STNi sample during joint annealing and alternatively changing the probing position in between the two samples. A photon energy of $E_{hv} = 680$ eV is used for the analysis.

Figure S7 depicts the fitting of the Ni 3p-Ti 3s core-level region in different sample states. Based on the Ni 3p-Ti 3s core-level region obtained from the as-prepared STNNi sample after an oxygen annealing step, the Ti 3s peak was fitted using a Voigt profile and the Ni 3p states (oxide component) were fitted using a Voigt doublet. For this purpose, the branching ratio of the Voigt doublet was fixed to the theoretical value for p-orbitals i.e. $1:2$ for $3p_{1/2} : 3p_{3/2}$. Subsequently, the Lorentzian and Gaussian widths for the Ti and Ni peaks as well as the magnitude of spin-orbit-splitting ΔBE$\sim$ 1.25 eV of the fitted Ni component was fixed. In addition, the peak position of the fitted Ti 3s component was fixed. Using the spectrum obtained from the reduced STNNi sample, a second Voigt doublet corresponding to the states of the metal nickel phase was fitted where a Lorentzian and Gaussian width and spin-orbit-splitting ΔBE equal to the oxide component was applied. Notably, the quality of fit decreases considerably when using a single Ni component (i.e. only one Voigt doublet) for the deconvolution of the Ni 3p-Ti 3s signature. Here, a second component is required to achieve a sufficient agreement of the fitting model with the experimental data obtained from reduced samples, particularly with respect to the intensity valley between the Ni 3p and Ti 3s core-levels. This observation is consistent with the evolution of a metallic Ni signal at lower binding energy relative to the oxide Ni component. The relative position of the fitted oxide and metal Ni 3p components was fixed and the resulting fitting model was applied to all spectra to evaluate the evolution of the peak area ratio. The fitting model was applied for the deconvolution of Ni 3p-Ti 3s spectra obtained from STNi accordingly.

Based on the peak fitting model, the surface Ni chemical evolution is analyzed by quantification of the different components on the basis of the respective peak area ratios (normalized to the Ti 3s component) as discussed in Fig. 3a, b of the main manuscript. As can be seen in Fig. S7c, the transient evolution of a metallic nanoparticle population, that is associated to considerable coarsening of the exsolved nanoparticles at the STNi surface results in a low intensity of the metallic signal across all sample states. For STNNi, the exsolution of a stable and finely dispersed nanoparticle population at the surface at an increased thermal reduction temperature of $T = 500°$C correlates with the detection of a pronounced metal signal (Fig. S7b). Note that the absolute binding energy

of the metal signal may slightly vary as it is influenced by the size of the supported nanoparticles as well as by interactions with the oxide support.[7–11]

Fig. S8 shows the total Ni enrichment at the surface of STNNi and STNi after different annealing steps. The plots corresponds to Fig. 3d e of the main manuscript, while denoting the respective share of the Ni_{oxide} and Ni_{metal} components contributing to the total amount of Ni. As can be seen, after thermal reduction at $T = 500°C$ a significant Ni_{metal} signal is only detected at the donor-type STNNi surface while a loss in the Ni_{metal} signal is apparent at the acceptor-type STNi surface.

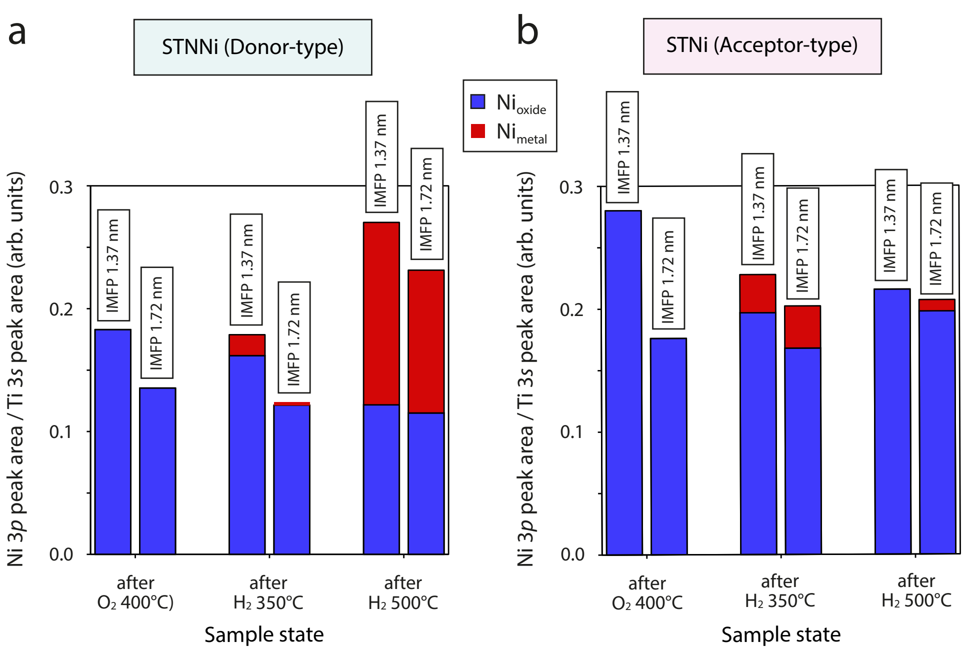

Figure S8: Ambient pressure x-ray photoelectron spectroscopy of STNNi and STNi. (a, b) Comparison of the total Ni $3p$ peak area normalized to the Ti $3s$ peak area after mild oxidation, low-temperature reduction at $T = 350°C$ and reduction at higher temperature of $T = 500°C$. A photon energy of $E_{hv} = 680\,eV$ and $E_{hv} = 900\,eV$ is applied for the variation of the information depth. Blue and red color denotes the respective share of the Ni_{oxide} and Ni_{metal} component.

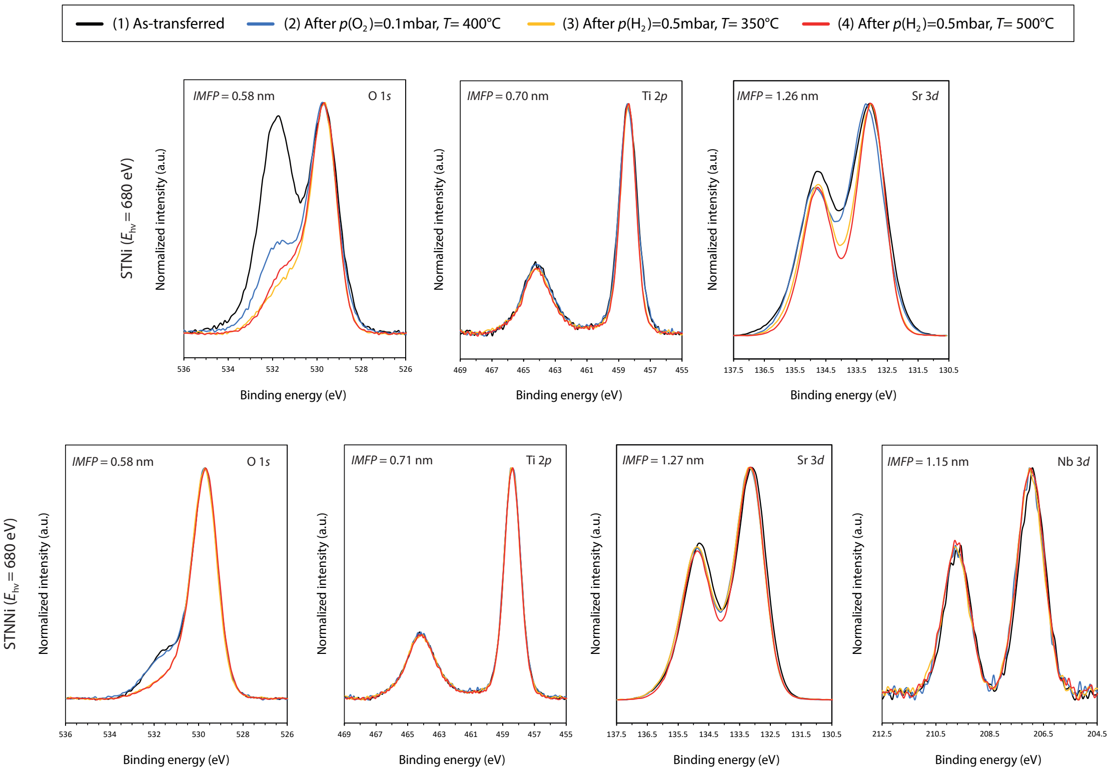

Figure S9: Ambient-pressure X-ray photoelectron spectroscopy. O 1*s*, Ti 2*p*, C 1*s*, Nb 3*d*, Sr 3*d* core-level spectra were periodically measured for (a) STNi and (b) STNNi. The measurements were performed under static conditions (UHV, room temperature) after consecutive annealing steps, corresponding to measurements 1, 2, 7 and 10 of the Ni 3*p*-Ti 3*s* core-level region shown in Fig. S7. A photon energy of $E_{\mathrm{hv}} = 680\,\mathrm{eV}$ was used for the analysis. The binding energy of all spectra were aligned with respect to the Ti 3*s* position and a Shirley-type background was subtracted. The spectra were obtained during joint annealing by alternately changing the probing position between the two samples.

Fig. S9 depicts superimposed XPS core-level spectra as periodically obtained from STNi (O 1*s*, Ti 2*p* and Sr 3*d*) and from STNNi (O 1*s*, Ti 2*p*, Sr 3*d* and Nb 3*d*) under static conditions (UHV, room temperature). The spectra were recorded from the as-transferred sample, after mild oxidation as well as after the first $T = 350°\mathrm{C}$ reduction step and after the second $T = 500°\mathrm{C}$ thermal reduction step. A photon energy of $E_{\mathrm{hv}} = 680\,\mathrm{eV}$ was used for the analysis. The IMFP for each of the core-level spectra is denoted in the figure.

For STNi thin films, pronounced changes are detected in the O 1*s* core-level spectrum. Two main peaks are apparent for the as-transferred sample. Here, the peak that is detected at a lower binding energy can be assigned to the perovskite lattice oxygen. A second peak of significant intensity is detected at higher binding energy, which is typically assigned to (mixed) hydroxides at the sur-

face of the oxide, where surface carbonates may further contribute to the slightly asymmetric peak shape. The strong affinity for surface hydroxylation upon exposure the atmosphere is in line with a large oxygen vacancy concentration at the surface of STNi[12,13]. No significant changes in in the spectra shape of the Ti $2p$ signature were detected in the different sample states. The Sr $3d$ spectrum exhibits variations in the relative weight and the intensity valley between the Sr $3d_{5/2}$ and the Sr $3d_{3/2}$ doublet, likely indicating the presence of a minor SrO or $Sr(OH)_2$ surface phase at the surface of the as-transferred sample as well as after mild oxidation of the thin films.

For STNNi thin films, only a small high-binding energy shoulder is detected in the O $1s$ core-level spectrum relative to the perovskite peak for as-transferred samples and for samples after mild oxidation. This observation may indicate a low affinity for surface hydroxylation related to a low oxygen vacancy concentration at the surface[12,13]. No significant changes in the Ti $2p$ and Nb $3d$ spectral shape are detected across the sample states. The evolution of more pronounced intensity valley in between the Sr $3d_{5/2}$ and the Sr $3d_{3/2}$ doublet becomes visible only after thermal reduction at $T = 500°C$, which may indicate the filling of strontium vacancies that are typically introduced by formation of a surface space charge region during material synthesis under oxidizing conditions[14]. Fig. S10 shows additional XPS and HR-STEM data to demonstrate that no significant Sr-rich phases form at the thin film surface or in the thin film bulk upon thermal reduction in the investigated time-temperature window. A comparison of the relative peak area ratios of A-site and B-site cations before and after thermal reduction are shown in Fig. S10a, b, respectively. As can be seen no significant changes in the respective A / B-ratio is detected for both STNNi (Fig. S10a) and STNi (Fig. S10b) after the thermal reduction treatment indicating that surface SrO formation as a consequence of B-site cation exsolution is negligible. Furthermore, HR-STEM imaging of the thin films in the as-prepared state and the thermally reduced state of representative STNNi and STNi samples is shown in (Fig. S10c-f). As can be seen, a coherent perovskite lattice is visible for all samples. We, therefore, can exclude degradation effects in form of phase segregation and the formation of extensive amounts of Sr-rich extended defects within the perovskite lattice. Based on our analysis, we hypothesize that the exsolution of B-site Ni dopants may be predominantly

compensated by the formation of Ruddlesden-Popper type defects (or to a lesser extent by the formation of B-site vacancies), as opposed to the decomposition of the perovskite structure, which limits the release of Sr cations.

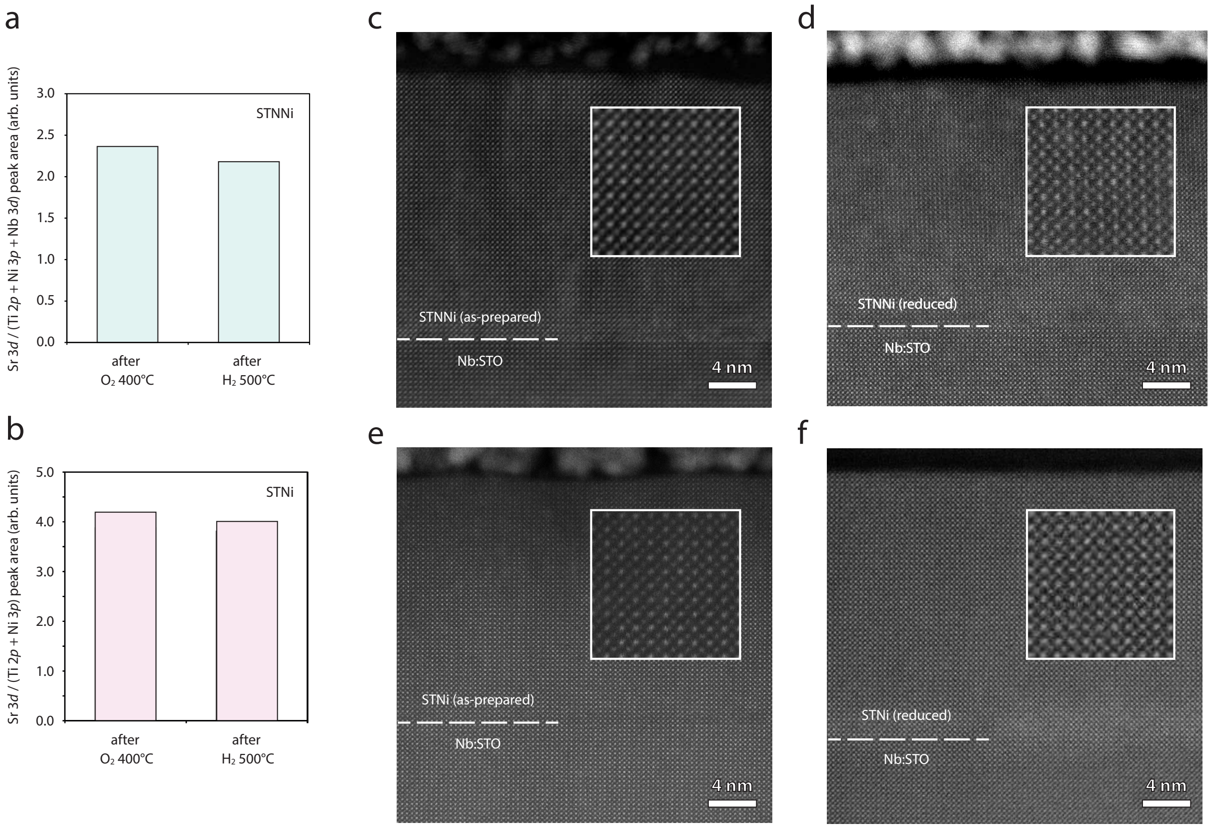

Figure S10: (a, b) Relative peak area ratio for A-site / B-site cations obtained from STNNi and STNi thin films after mild oxidation and after thermal reduction at $T = 500°C$ using a photon energy of $E_{hv} = 680$ eV for the analysis, respectively. (c-f) High resolution transmission electron microscopy images comparing STNNi and STNi thin films in the as-prepared state and after thermal reduction. The inset images show a magnification of $\sim$10x10 unit cells, each obtained from the main images.

Supplementary Note 5: Dewetting of sputtered thin films

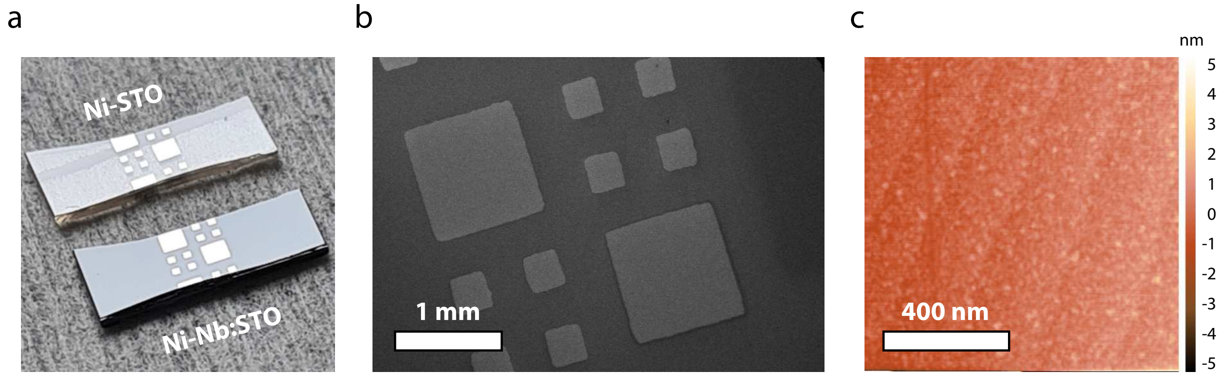

Figure S11: As-prepared 20 nm thick Ni thin films prepared by sputtering on TiO_2-terminated STO and Nb:STO single-crystal substrates. (a) Photograph of pieces of Ni-STO and Ni-Nb:STO cut from a 10 x 10 mm sample. (b) SEM imaging of the patterned thin film. (c) AFM imaging of the surface morphology obtained in the center of one of the large Ni pads shown in (b).

Fig. S11 shows the characterization of sputtered Ni thin films used for dewetting experiments as discussed in the main manuscript. Dense Ni thin films of 20 nm were sputtered on the surface of TiO_2-terminated STO and Nb:STO substrates using a shadow mask. A defined square patterned thin film was obtained, where the thin film surface exhibits a smooth surface morphology. The sputtered Ni thin films are employed for quasi-Winterbottom analysis after dewetting under reducing conditions as discussed in the main manuscript.

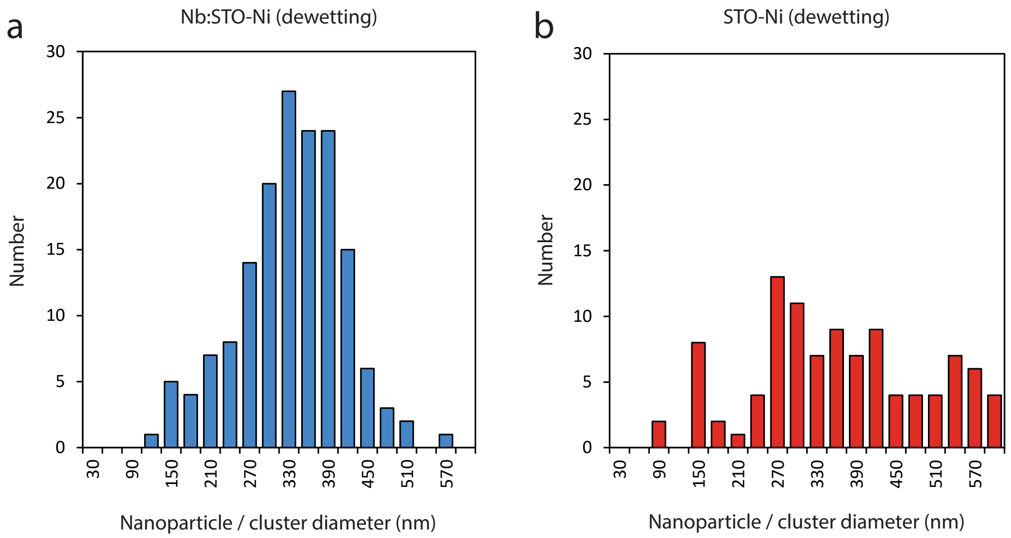

Figure S12: Frequency distribution of the nanoparticle / cluster diameter of Ni nanoparticles fabricated by dewetting of a sputtered Ni thin film on a Nb:STO and a STO single crystal substrate ($T_1 = 1000$°C, $t_1 = 5$ h and $T_2 = 1050$°C, $t_2 = 23$ h in p(4% H_2/Ar) = 1 bar). The particle / cluster diameters are determined based on scanning electron microscopy, using an average of three measurements for each particle, respectively.

The size distributions for Ni nanoparticles, fabricated by dewetting of sputtered Ni thin films, corresponding to Fig. 4 of the main manuscript is shown in Fig. S12. As can be seen, a unimodal frequency distribution is detected for Ni particles on the donor-type Nb:STO substrate, while a non-normal frequency distribution becomes apparent for Ni particles fabricated on the impurity acceptor-doped STO substrate. Here, an increased number of large Ni particles / clusters points towards pronounced coalescence effects. Notably, the increased coalescence effects during dewetting of Ni on STO, are correlated to a lower nanoparticle density of 1.2 μm^{-2} (versus 1.7 μm^{-2} for dewetting of Ni on Nb:STO).

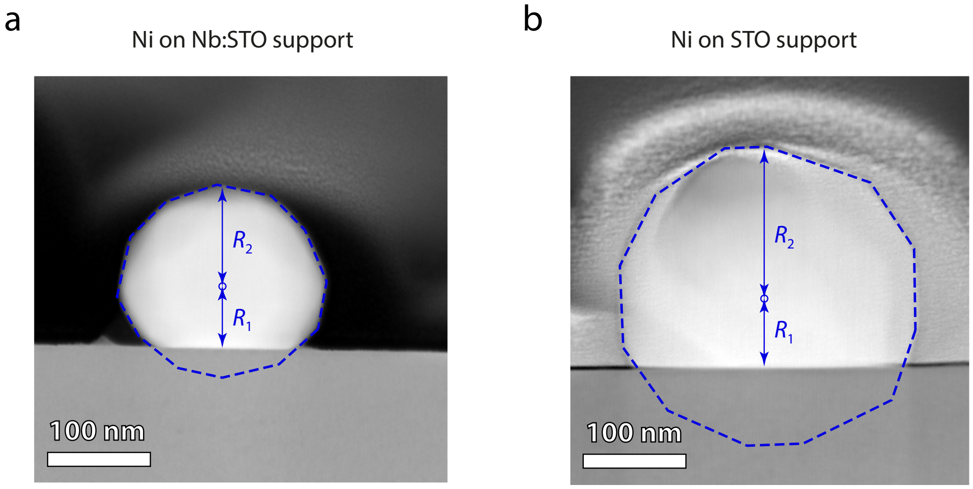

Figure S13: Scanning transmission electron microscopy of the nanoparticle-support interface of samples that were synthesized by dewetting of 20 nm thick Ni thin films that were sputtered on (001) Nb:STO and STO substrates ($T_1 = 1000$°C, $t_1 = 5$ h and $T_2 = 1050$°C, $t_2 = 23$ h in p(4% H_2/Ar) = 1 bar). Representative cross-section imaging of Ni a nanoparticles on (a) an (donor-type) Nb:STO and a acceptor-type STO substrate. The approcimate perimeter of the particles is highlighted by dashes blue lines, the Wulff center is denoted by a blue circle and R_1 / R_2 determined from the images and used for quasi-Winterbottom analysis are indicated by blue arrows.

A quasi-Winterbottom analysis is performed to investigate differences in the wetting behaviour of metallic Ni particles synthesized by dewetting of sputtered Ni thin films on donor-type Nb:STO (Fig. S13a) and acceptor-type STO (Fig. S13b) substrates under reducing conditions ($T_1 = 1000$°C, $t_1 = 5$ h and $T_2 = 1050$°C, $t_2 = 23$ h in p(4% H_2/Ar) = 1 bar). The particles were found to be aligned in different orientations (e.g. (001), (011) and (111) with respect to the (001)-oriented substrate) or misaligned with respect to the crystal lattice of the substrate, which may indicate that the particles have not yet reached local equilibrium with the respective substrate under the given annealing conditions (a true Winterbottom analysis requires all particles being present in equilibrium shape[15]). However, relative differences in the interfacial energy between the Ni-Nb:STO and the Ni-STO material systems can be compared on the basis of the wetting behaviour at the particle-substrate interface. Notably, the dewetted thin films have experienced much harsher conditions than the exsolved nanoparticles at the STNNi and STNi surface. The relative distance from the center of the Wulff shape of the nanoparticle to the particle-substrate interface is determined, where R_1 is the distance from the particle-substrate interface to the Wulff center, and R_2 is the distance from the Wulff center to the uppermost facet of the particle (Fig. S13).

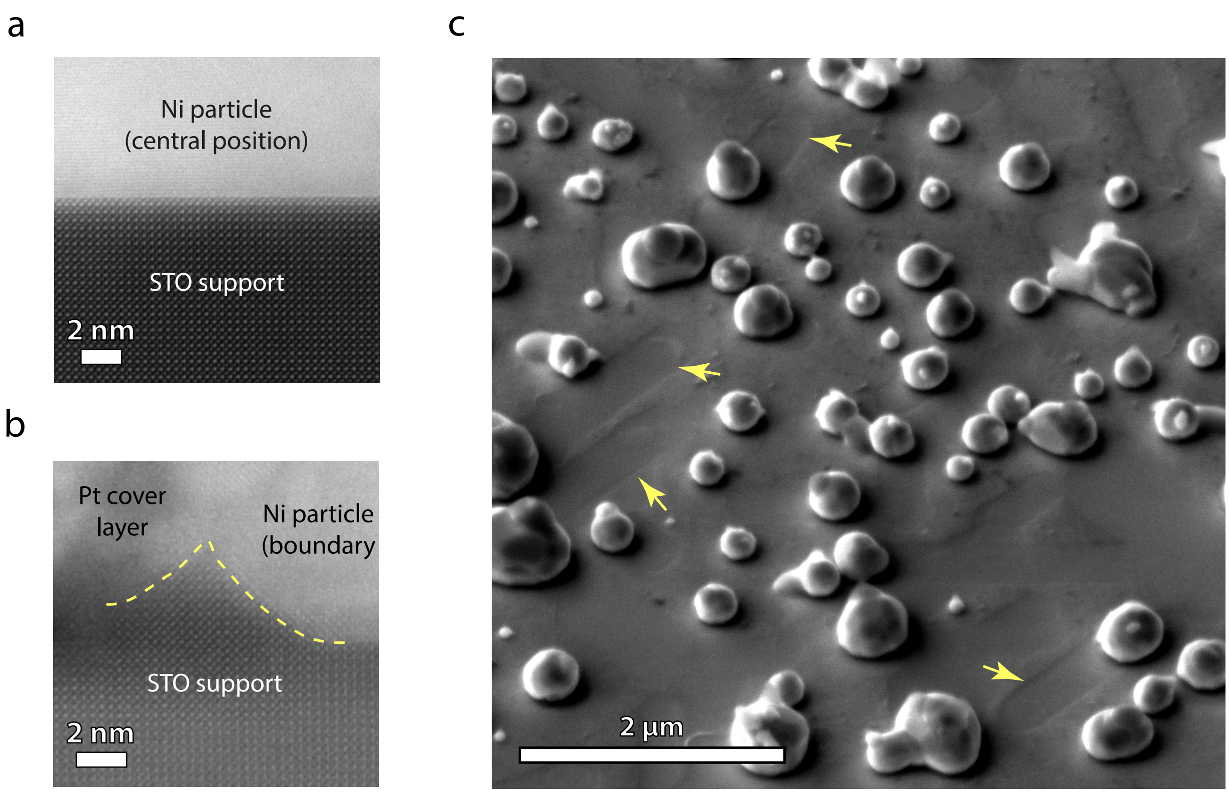

Figure S14: Scanning electron microscopy and Scanning transmission electron microscopy of the nanoparticle-support interface of Ni nanoparticles that were synthesized by dewetting on SrTiO3.(a) HR-STEM imaging of the central particle-support interface showing a defined interface of nanoparticles synthesized by dewetting on the oxide substrates. (b) HR-STEM imaging of the particle boundary, indicating the formation of a base structure (highlighted by yellow line) at the particle-support interface after dewetting that may be interpreted as an early stage of nanoparticle socketing. (c) SEM imaging of Ni nanoparticles on an $SrTiO_3$ substrate synthesized by dewetting of a sputtered 20 nm thick Ni thin film, respectively ($T_1 = 1000$°C, $t_1 = 5$ h and $T_2 = 1050$°C, $t_2 = 23$ h in p(4% H_2/Ar) = 1 bar). The formation of ‚trails' is associated with the formation of the supported nanoparticles on the substrate is visible. Several trails are highlighted by yellow arrows.

Dewetting of a sputtered Ni thin film on a $SrTiO_3$ under reducing atmosphere, results in the formation of nanoparticles with inhomogeneous spatial and size distribution (Fig. S14). In Fig. S14a a Z-contrast image recorded at the center of the Ni-STO interface shows a Ni particle sitting on the B-site terminated STO substrate. For comparison an image recorded at the surface boundary of the particle is shown in Fig. S14b, where the formation of a ridge becomes apparent. A recent study on exsolution catalysts has shown that nanoparticle socketing is not directly associated to the exsolution process but proceeds in a subsequent modification of the metal-oxide interface as described above[16]. Our study shows similar topological features forming at the oxide support after high-temperature dewetting of a sputtered Ni thin films. It is worth noting that our imaging

study has revealed that such ridges or sockets are often present in the vicinity of the supported nanoparticles, while however, being frequently detached from the particles. Furthermore, the formation of ‚trails', i.e. topological features in close proximity to many of the particles synthesized by dewetting is visible on the STO substrate (Fig. S14c). Exemplarily, prominent trail features are highlighted by yellow arrows in Fig. S14c. In a recent study, the formation of trails proceeding from nanoparticles synthesized by metal exsolution was demonstrated and associated to the mobility of nanoparticles at elevated temperatures [17]. The formation of these topological features may be related to the formation of ridges [16] and sockets [16,18], which were shown to accompany metal exsolution reactions. The evolution of these structural surface features may have a mutual origin, while they appear to have no significant influence on the thermal stability, i.e. the growth and coalescence of the supported Ni nanoparticles in the present material system.

Considering these findings it is important to emphasize that it is very likely that nanoparticle socketing is not a unique feature of metal exsolution and hence not limited to exsolved nanoparticles but rather is related to the nature of the equilibrated metal-oxide interface as shown in another recent study [17]. Therefore, it can be hypothesized that the extent of the particle embedment may be specific for the nature of the respective metal-oxide material system. Here, further research is needed to identify the role of defects, such as A-site deficiency which is commonly introduced in exsolution-active perovskite parents [19], for the formation of nanoparticle sockets.

Supplementary Note 6: Exsolution under humid conditions

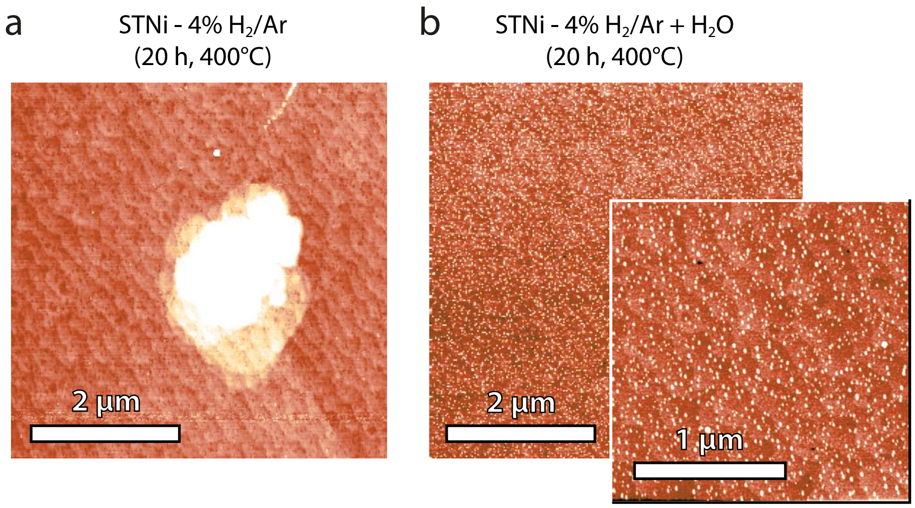

Figure S15: Comparison of the surface morphology of STNi after thermal reduction in (a) dry conditions ($T = 400°C$, $t = 20$ h, $p(4\%\ H_2/Ar) = 1$ bar) and (b) humid conditions ($T = 400°C$, $t = 20$ h, $p(4\%\ H_2/Ar/H_2O) = 1$ bar).

In Fig. S15, the exsolution behavior of STNi is compared after thermal reduction in dry conditions (Fig. S15a) and in humid conditions (Fig. S15b). Notably, dry reduction conditions are employed for all measurements throughout the main paper leading to fast coalescence of exsolved Ni nanoparticles (cf. Fig. S4 for further comparison). For humidification of the gas mixture the 4% H_2/Ar gas flow was passed through a water bath at room temperature before entering the quartz tube of the quench furnace. As can be seen, the exsolution behaviour changes dramatically, where pronounced coarsening is visible for dry conditions and finely dispersed nanoparticles decorate the acceptor-type STNi surface after thermal reduction in a humidified gas atmosphere.

References

(1) Weber, M. L. *et al.* Reversibility limitations of metal exsolution reactions in niobium and nickel co-doped strontium titanate. *Journal of Materials Chemistry A* **11**, 17718-17727 (2023).

(2) Weber, M. L. *et al.* Exsolution of embedded nanoparticles in defect engineered perovskite layers. *ACS nano* **15**, 4546–4560 (2021).

(3) Baeumer, C. *et al.* Surface termination conversion during $SrTiO_3$ thin film growth revealed by x-ray photoelectron spectroscopy. *Scientific reports* **5**, 11829 (2015).

(4) Yan, Hong, et al. Stoichiometry and termination control of $LaAlO_3/SrTiO_3$ bilayer interfaces. *Advanced materials interfaces* **8**, 2001477 (2021).

(5) Lei, Qingyu et al. Constructing oxide interfaces and heterostructures by atomic layer-by-layer laser molecular beam epitaxy. *npj Quantum Materials* **2**, 10 (2017).

(6) Sun, H. Y. et al. Chemically specific termination control of oxide interfaces via layer-by-layer mean inner potential engineering. *Nature communications* **9**, 2965 (2018).

(7) Mason, M. G. Electronic structure of supported small metal clusters. *Physical review B* 27, 748–762 (1983).

(8) Wertheim, G. K. & DiCenzo, S. B. Cluster growth and core-electron binding energies in supported metal clusters. *Physical review B* **37**, 844–847 (1988).

(9) Wertheim, G. K. Electronic structure of metal clusters. *Small Particles and Inorganic Clusters*, 319-326 (1989).

(10) Richter, Bodo et al. Cluster core-level binding-energy shifts: the role of lattice strain. *Physical review letters* **93**, 026805 (2004).

(11) Jiang, Zhiquan et al. Direct XPS evidence for charge transfer from a reduced rutile TiO_2 (110) surface to Au clusters. *The Journal of Physical Chemistry C* **111**, 12434-12439 (2007)

(12) Mefford, J. T., Hardin, W. G., Dai, S., Johnston, K. P. & Stevenson, K. J. Anion charge storage through oxygen intercalation in $LaMnO_3$ perovskite pseudocapacitor electrodes. *Nature Materials* **13**, 726–732 (2014).

(13) Stoerzinger, K. A. et al. Speciation and electronic structure of $La_{1-x}Sr_xCoO_{3-\delta}$ during oxygen electrolysis. *Topics in Catalysis* **61**, 2161-2174 (2018).

(14) Weber, M. L. et al. Space charge governs the kinetics of metal exsolution. *Nature Materials* **23**, 406–413 (2024).

(15) Winterbottom, W. Equilibrium shape of a small particle in contact with a foreign substrate. *Acta Metallurgica* **15**, 303–310 (1967).

(16) Calì, E. et al. Real-time insight into the multistage mechanism of nanoparticle exsolution from a perovskite host surface. *Nature communications* **14**, 1754 (2023).

(17) Jennings, D., Ricote, S., Santiso, J., Caicedo, J. & Reimanis, I. Effects of exsolution on the stability and morphology of Ni nanoparticles on BZY thin films. *Acta Materialia* **228**, 117752 (2022).

(18) Neagu, D. et al. Nano-socketed nickel particles with enhanced coking resistance grown in situ by redox exsolution. *Nature communications* **6**, 8120 (2015).

(19) Neagu, D., Tsekouras, G., Miller, D. N., Ménard, H. & Irvine, J. T. S. In situ growth of nanoparticles through control of non-stoichiometry. *Nature chemistry* **5**, 916–923 (2013).
